# Influence of climatic variables on the *Aedes aegypti* and *Culex quinquefasciatus* populations in Mato Grosso, Brazil

**DOI:** 10.1590/0037-8682-0185-2019

**Published:** 2020-03-16

**Authors:** Lucinéia Claudia De Toni Aquino da Cruz, Alexander Gonçalves Ferreira Guimarães, Emerson Marques de Souza, Raquel da Silva Ferreira, Raphael de Souza Rosa Gomes, Renata Dezengrini Slhessarenko, Marina Atanaka

**Affiliations:** 1 Universidade Federal de Mato Grosso, Programa de Pós-graduação em Saúde Coletiva, Cuiabá, MT, Brasil.; 2 Universidade Federal de Mato Grosso, Programa de Pós-graduação em Biociências, Cuiabá, MT, Brasil.; 3 Universidade Federal de Mato Grosso, Programa de Pós-graduação em Física Ambiental, Cuiabá, MT, Brasil.; 4 Universidade Federal de Mato Grosso, Programa de Pós-graduação em Ciências da Saúde, Cuiabá, MT, Brasil.

**Keywords:** Aedes aegypti, Climatic variables, Culex quinquefasciatus

## Abstract

**INTRODUCTION:**

*Aedes aegypti* and *Culex quinquefasciatus* are vector species responsible for the transmission of important arboviruses.

**METHODS:**

Adult mosquitoes were collected in the urban areas of four municipalities in Mato Grosso within 1 year.

**RESULTS:**

A total of 19,110 mosquitoes were collected. Among them, 16,578 (86,8%) were *C. quinquefasciatus* (44% female and 56% male); 2,483 (13%), *A.* (Stegomyia) *aegypti* (54% female and 46% male); and 49 (0,30%), from the genus Psorophora, Anopheles, Coquilettidia, and Sabethes. A significant correlation was observed between the number of mosquitoes from all species and dew point (female mosquitoes, p = 0.001; male mosquitoes, p = 0.001).

**CONCLUSIONS:**

The results of this study may be used as environmental indicators of mosquito populations.

Some insects, such as *Aedes aegypti*, which causes dengue, Zika virus, and Chikungunya virus, and *A.* (Stegomyia) *aegypti*, which transmits yellow fever, are responsible for epidemics that have a major seasonal impact on public health. *Culex quinquefasciatus* is a highly anthropophilic species, abundant in urban areas, and considered a secondary vector of arboviruses, such as Oropouche fever, Saint Louis encephalitis, and Zika virus^1^. Both *A.* (Stegomyia) *aegypti* and *C. quinquefasciatus* have been implicated in the experimental transmission of Mayaro virus^2^. 

Among the factors that contribute to vector proliferation, climatic variables have been widely discussed because they interfere with ecological balance and consequently with the intensity of vector infestation indices^3^. Previous studies have shown that the impact of climate variations and changes in the life cycle of vectors are associated with the occurrence of epidemics^3^. 

The climate conditions in Brazil are highly favorable for vector proliferation and consequently the introduction and dissemination of arboviruses. The state of Mato Grosso has the highest incidence of Chikungunya virus (100.3 cases per 100,000 inhabitants) within the Central-Western region; meanwhile, the incidence rates of Zika virus and dengue are 63.3 and 268.1 cases per 100,000 inhabitants, respectively. Moreover, cases of Mayaro virus and Oropouche fever in the state have been previously reported^4^. Evidence of the circulation of West Nile virus; Ilhéus, Rocio, and Saint Louis encephalitis virus; and Eastern, Western, and Venezuelan equine encephalitis virus has been occasionally observed in Mato Grosso and Pantanal^5^.

Considering the favorable environmental conditions for the proliferation of vector populations and the studies showing the relationship between climatic factors and the dynamics of arboviruses, the present study aimed to assess the correlation between the presence and infestation of vectors as well as climatic variables in the municipalities in the state of Mato Grosso in 2017.

The urban areas chosen for vector monitoring in the state of Mato Grosso were Cuiabá (capital), Rondonópolis, Sinop, and Cáceres (Figure 1). These places were included in the analysis because they are of great epidemiological importance and have the largest human populations in the state and high rates of vector infestation and consequently high cases of arboviruses annually.

**FIGURE 1:**
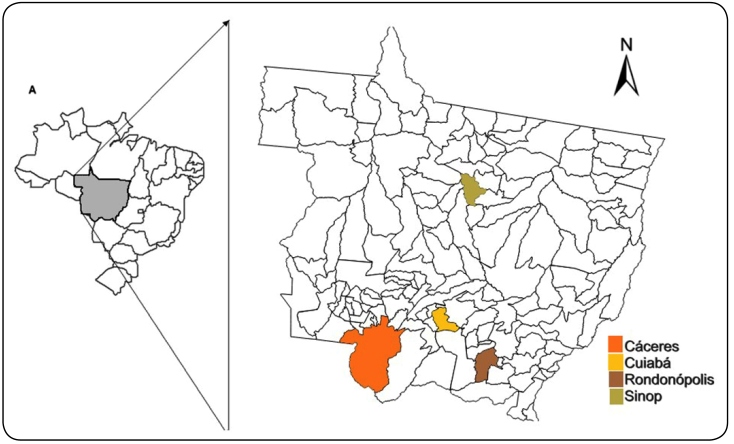
Location of the state of Mato Grosso, and the municipalities where the samples of mosquitoes and environmental variables were collected.

Adult insects were captured with Nasci aspirators between February 2017 and January 2018 every 2 months in each municipality. Based on a previous study carried out in the state that had a 0.5% margin of error and used the *A.* (Stegomyia) *aegypti* rapid index criteria established by the Ministry of Health, we used the proportional estimation method in infinite populations to calculate the number of houses that should be sampled in each of the four municipalities. 

The sample comprised 2,132 properties distributed proportionally in the selected neighborhoods in the four municipalities. The properties were chosen via random selection, with an average of 12 blocks per neighborhood with 3-5 residences in each block. The first property in the block was selected in a clockwise shift manner. This procedure allowed the replacement of a residence by another one located right after whenever possible if it was close or if the residents refused to participate.

Mosquito samples were collected between 8:00 and 11:00 h and 14:00 and 17:00 h in the outdoor and indoor areas of residencies owned by individuals who signed the consent form. A researcher and an endemic control agent assigned in the area visited all residences for 20 min. The study was previously approved by the Ethics Committee Research at the Julio Muller University Hospital - (number: 1.689.683). 

The dependent variable was the number of insects collected (*A.* [Stegomyia] *aegypti* and *C. quinquefasciatus*). Some variables included only mosquito females, as these are an important indicator of transmission.

Other variables that were assessed included collection sites (municipalities and neighborhoods), seasonality correlated to the months when collection was performed, classified as the rainy and dry seasons, and microclimatic variables. Data about climatic variables, including room temperature, dew point temperature, thermal amplitude, relative humidity, atmospheric pressure, wind speed, ultraviolet (UV) index, and rain index, were obtained locally with the Weather Underground application and were compared with those from the National Meteorological Institute. Descriptive and variance analyses (ANOVA; R software version 3.5) were conducted, and kernel intensity and location maps with 500 m (QGIS 2.18.25) were established.

Of the 19,111 mosquito specimens, 16,578 (86,8%) were *C. quinquefasciatus* (44% female and 56% male); 2,483 (13%), *A.* (Stegomyia) *aegypti* (54% female and 46% male); and 49 (0,30%), from the genus Psorophora*,* Anopheles*,* Coquillettidia, and Sabethes. Caceres (6,057; 32%) had the highest number of mosquitoes captured, followed by Cuiabá (5,607; 29%), Rondonópolis (4,338; 23%), and Sinop (3,108; 16%). 

Figure 2 shows the total number of mosquitoes captured and the mean climatic variables during each collection. When analyzing the correlation between the total number of insects and climatic variables, independent of city and neighborhood, these variables had a direct influence on the distribution and presence of mosquitoes (*p* = 0.0028). 

**FIGURE 2:**
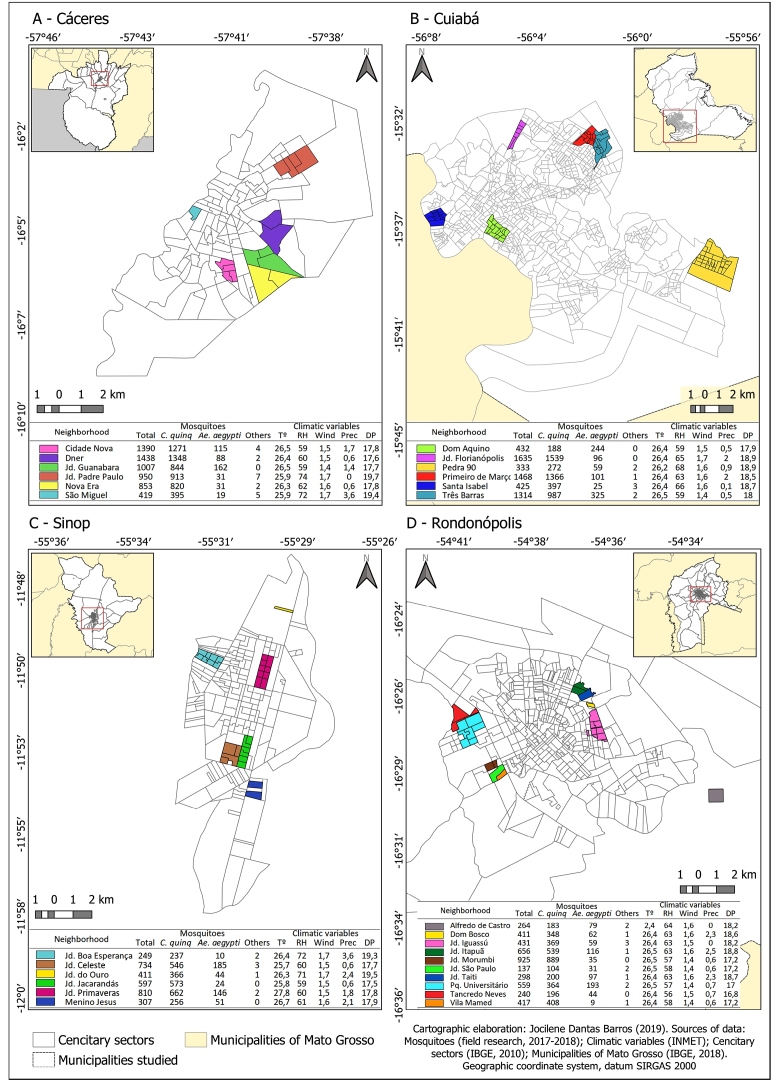
Spatial distribution of mosquitoes and climatic variables according to neighborhood and municipality: **(A)** Cáceres, **(B)** Cuiabá, **(C)** Rondonópolis, and **(D)** Sinop. **T (°):** temperature; **RH:** relative humidity; **Prec:** precipitation; **DP:** dew point temperature.

Among the variables, the variable temperature of dew point was highly significant for all mosquito species. Moreover, when analyzed separately, in addition to dew point temperature, relative humidity (*p* = 0.0001) and wind velocity were significantly associated with the number of *A.* (Stegomyia) *aegypti* (female, *p* = 0.007; male, *p* = 0.004). 

During the collection period, the presence of mosquitoes differed in terms of seasonality. A more significant correlation (p = 7.06E-07) was observed between rainy season and the number of *A.* (Stegomyia) *aegypti* (rainy season: n = 1,586; dry season: n = 895) (Figure 3). Rainy season and the number of female *A.* (Stegomyia) *aegypti* were also associated (female, *p* = 2.13E-06; male, *p* = 0.0001). The density of female *A.* (Stegomyia) *aegypti* is an important epidemiological indicator.

**FIGURE 3:**
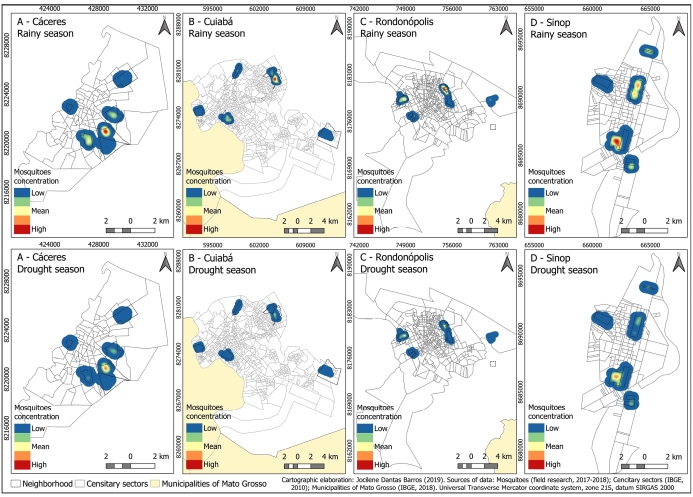
Spatial distribution of the *Aedes aegypti* species between the rainy and dry periods.

The analysis of climatic variables according to the city that was sampled and independent of neighborhood revealed that dew point and relative humidity (both with *p* = 0.008) and wind velocity (*p* = 0.009) influenced the total number of mosquitoes captured in Cáceres.

In Cuiabá, the variables significantly associated with the total number of mosquitoes included a mean wind velocity of 1.77 m/s (*p* = 4.01556E-05), atmospheric pressure, and seasonality (*p* = 0.009). As in Cáceres, the dew point temperature in Rondonópolis ranged from 10ºC to 26ºC (*p* = 0.004). In Sinop, none of the variables were significantly correlated to the total number of mosquitoes captured. Specifically, the association between the abundance of *A.* (Stegomyia) *aegypti* and some climatic variables significantly differed according to municipality: dew point temperature in Cáceres, Cuiabá, and Rondonópolis (*p* = 0.001, *p* = 0.003, and *p* = 0.002, respectively). Rainy season was significantly associated with the total number of mosquitoes in Cáceres (*p* = 9.9873E-06), Cuiabá (*p* = 0.001), and Rondonópolis (*p* = 0.001). Meanwhile, wind velocity was associated with the total number of mosquitoes in Cáceres (*p* = 0.007) and Cuiabá (*p* = 2.54064E-05). Meanwhile, relative humidity was significantly correlated to the total number of mosquitoes only in Cuiabá (*p* = 1.45853E-05). 

Notably, UV index was correlated to the number of female *C. quinquefasciatus* only in Sinop (*p* = 0.002).

Climatic variables were not significantly correlated to the total number of female *A.* (Stegomyia) *aegypti* in the neighborhoods of Cáceres only. In the neighborhood of Dom Aquino in Cuiabá, relative humidity (*p* = 0.0003), wind velocity (*p* = 0.0001), and atmospheric pressure (*p* = 0.0011) were significantly associated with the total number of mosquitoes. In addition, climatic variables, such as dew point temperature and relative air humidity, were significantly correlated to the total number of mosquitoes and *Culex* specimens in the neighborhoods of Santa Isabel, Três Barras, and Jardim Florianópolis in Cuiabá. In the neighborhood of Dom Bosco in Rondonópolis, a significant association was observed between the total number of mosquitoes as well as wind velocity (*p* = 0.0002), rainfall index (*p* = 0.004), dew point temperature (*p* = 0.0014), atmospheric pressure (*p* = 0.0003), and relative humidity (*p* = 0.0003). Meanwhile, in the neighborhoods of Jardim São Paulo, Jardim Taiti, and Alfredo de Castro in Rondonópolis, a significant correlation was observed between the total number of mosquitoes as well as UV index (*p* = 0.0064), dew point temperature (*p* = 0.003), and environmental temperature (*p* = 0.053). In the neighborhood of Jardim Primaveras in Sinop, a significant correlation was observed between the number of female *A.* (Stegomyia) *aegypti* and dew point temperature (*p* = 0.00008). In the neighborhood of Jardim Jacarandás in Sinop, a correlation was noted between the number of male *C. quinquefasciatus* and relative humidity (*p* = 0.008). 

In the neighborhood of Jardim Padre Paulo in Cáceres, a significant correlation was observed between the total number of mosquitoes and relative humidity, dew point, atmospheric pressure, and seasonality (*p* = 0.005). Radiation was significantly correlated to the number of male *A.* (Stegomyia) *aegypti* (*p* = 0.006) in the neighborhoods of DNER and Nova Era (*p* = 0.0009); in this place, relative humidity (*p* = 0.001), dew point (*p* = 0.001), and seasonality (*p* = 0.005) were also significantly associated with the total number of mosquitoes.

This study focusing on populous and endemic urban areas for arboviruses showed that specific climatic variables are strongly correlated to the presence and abundance of mosquitos in Mato Grosso. 

Dew point temperature was significantly associated with the number of mosquito species captured in Cáceres and Rondonópolis. This variable corresponds to the amount of water vapor condensed in the air, which may influence the density of mosquitoes and hatching of insect eggs. A similar result was described by Silva et al. (2018)^6^ in Rio de Janeiro and by Gleiser and Gorla (2007)^7^ in Argentina. That is, dew point temperature was significantly associated with the distribution of vectors of arbovirus, which is responsible for encephalitis. De Groote et al. (2008)^8^ have shown that this variable was significantly associated with the occurrence of different *Culex* species responsible for the transmission of the West Nile virus (WNV) in the United States. 

Relative humidity and wind velocity were specifically correlated to the number of female *A.* (Stegomyia) *aegypti* in Cáceres regardless of neighborhood. These factors influence the level of activity of different mosquito species. Canyon et al. (2013)^9^ have reported that *A.* (Stegomyia) *aegypti* remains active under experimentally diminished humidity conditions without influencing sting rate. 

Seasonality is the condition that has the most significant influence on entomological parameters, such as mortality rates, development, and oviposition. Our study showed that a higher number of mosquitoes is observed in the rainy season, with significant data obtained in Cuiabá, as shown in other studies^10^. Moreover, as in Sinop, a significant correlation was observed between the total number of mosquitoes and atmospheric pressure in Cuiabá. This variable influences entry into shelters as insects can detect differences in climate based on changes in atmospheric pressure. A similar result was described in a study conducted in Rio de Janeiro^11^. 

Notably, the present analysis showed a significant correlation between UV index and the number of *C. quinquefasciatus* in Sinop, but not in other regions. 

The areas in Mato Grosso that were assessed presented with different patterns of urban space organization, which has a direct influence on microclimate. In Sinop, a correlation was observed between dew point and the number of *A.* (Stegomyia) *aegypti* in Jardim Primavera and the number of *C. quinquefasciatus* in the neighborhood of Jardim Jacarandás; both regions are adjacent to areas with greater vegetation coverage, as represented by the Cerrado-Amazon transition forests^12^. The degree of vegetation coverage influences the distribution of *A.* (Stegomyia) *aegypti* and *A.* (Stegomyia) *albopictus* in previous studies^13^. This index helps reduce temperature and decrease the so-called heat islands, both via interception of solar rays and via air cooling during the evapotranspiration process, thereby affecting the levels of mosquito infestation^13^.

The neighborhood of Dom Aquino in Cuiabá, where a correlation between climatic variables and the number of female *A.* (Stegomyia) *aegypti* was observed, was woodier than most sites that were visited. The impact of temperature as a variable was not observed. However, shading might had influenced humidity levels, as observed by Prado et al. in Espirito Santo, Brazil^14^.

In the neighborhoods of Cáceres, climatic variables were correlated to the presence of *A.* (Stegomyia) *aegypti* and *C. quinquefasciatus.* Moreover, these sites have a thicker vegetation and shading with peculiar environmental characteristics due to its geographic location that is adjacent to Pantanal, which has a biological diversity associated with multiple and complex environmental interactions^15^. 

The neighborhood Nova Era in Cáceres, which presented a correlation for *A.* (Stegomyia) *aegypti*, have sparse sanitation services and water supply. Although these variables were not included in the analyses, they are known to have an influence on mosquito population. 

Considering that vector populations are conditioned by ecosystem dynamics, presence of niches for adaptation and microclimate variations may favor arbovirus circulation and expansion to naive areas. Taken together, the results of this study underscored the vast diversity in the state of Mato Grosso, which has a unique geographic confluence of Pantanal wetland in the South and East, Cerrado, and Amazon transitions in the North and East, and conditions with a great environmental complexity that alters climatic variables influencing the distribution and abundance of vector population according to seasonality^15^. The uniqueness of the environmental variables identified in each municipality may help in the identification of important surveillance parameters for the establishment of control measures against arbovirus epidemic.
